# Design Factors of Ti-Base Abutments Related to the Biomechanics Behavior of Dental Implant Prostheses: Finite Element Analysis and Validation via In Vitro Load Creeping Tests

**DOI:** 10.3390/ma17153746

**Published:** 2024-07-29

**Authors:** Jordi Martínez-Grau, Daniel Robles, Román A. Pérez, Xavier Marimon, Saray Fernández-Hernández, Carlos Aroso, Aritza Brizuela-Velasco

**Affiliations:** 1 Bioengineering Institute of Technology (BIT), Universitat Internaciional de Catalunya (UIC), 08195 Sant Cugat del Vallés, Spain; jordi.martinezg@soadco.com (J.M.-G.); rperezan@uic.es (R.A.P.); xmarimon@uic.es (X.M.); 2 DENS-ia Research Group, Faculty of Health Sciences, Miguel de Cervantes European University, 47012 Valladolid, Spain; sfernandezh@uemc.es (S.F.-H.); abrizuela@uemc.es (A.B.-V.); 3 UNIPRO—Oral Pathology and Rehabilitation Research Unit, University Institute of Health Sciences (IUCS), Cooperativa de Ensino Politécnico e Universitario (CESPU), 4585-116 Granda, Portugal; carlos.ribeiro@iucs.cespu.pt

**Keywords:** finite element analysis, Ti base, abutments, dental implants, stress distribution, strain distribution

## Abstract

This study has been carried out to analyze the influence of the design of three geometric elements (wall thickness, platform width, and chamfer) of Ti-base abutments on the distribution of stresses and strains on the implant, the retention screw, the Ti base, and the bone. This study was carried out using FEA, analyzing eight different Ti-base models based on combinations of the geometric factors under study. The model was adapted to the standard Dynamic Loading Test For Endosseous Dental Implants. A force of 360 N with a direction of 30° was simulated and the maximum load values were calculated for each model, which are related to a result higher than the proportional elastic limit of the implant. The transferred stresses according to von Mises and microdeformations were measured for all the alloplastic elements and the simulated support bone, respectively. These results were validated with a static load test using a creep testing machine. The results show that the design factors involved with the most appropriate stress distribution are the chamfer, a thick wall, and a narrow platform. A greater thickness (0.4 mm) is also related to lower stress values according to von Mises at the level of the retaining screws. In general, the distributions of tension at the implants and microdeformation at the level of the cortical and trabecular bone are similar in all study models. The in vitro study on a Ti-base control model determined that the maximum load before the mechanical failure of the implant is 360 N, in accordance with the results obtained for all the Ti-base designs analyzed in the FEA. The results of this FEA study show that modifications to the Ti-base design influence the biomechanical behavior and, ultimately, the way in which tension is transferred to the entire prosthesis–implant–bone system.

## 1. Introduction

Computer-aided design (CAD) and computer-aided manufacturing (CAM) technologies have become the most widely used manufacturing methods in oral rehabilitation and applied both to conventional fixed prostheses and to implants. The application of this digital workflow brings numerous advantages, among which are a shorter production time, a higher level of customization and precision, and a greater number of materials to choose from [1]. Within the CAM processes, two types can be considered: subtractive CAM, which refers to computer numeric controlled milling of a desired shape out of a prefabricated material block, and additive CAM, which in turn refers to three-dimensional printing and is a process in which the desired object is produced by the deposition of layer upon layer. Although subtractive CAM is considered the gold standard, from an engineering point of view, additive CAM has the advantage of overcoming the geometric restrictions present with subtractive CAM, more complex forms may be produced, and the waste of material may be reduced [2].

The most prevalent material established through subtractive CAM technology is zirconium oxide (zirconia). It is a material of high biocompatibility, suitable mechanical properties, and even adequate esthetics [3]; these last two properties allow it to be used as a monolithic restorative material (at total thickness). However, due to its crystalline structural properties, zirconia is relatively weak before the application of shear loads and, in addition, has different elastic properties from titanium, from which oral implants are commonly manufactured (Young’s modulus of 200 versus 107 Gpa, respectively). Therefore, although it is possible to mill complete zirconia prostheses, including the internal connection of the implant, there is a risk of suffering a technical complication of fracture at this level [4].

**Figure 1:**
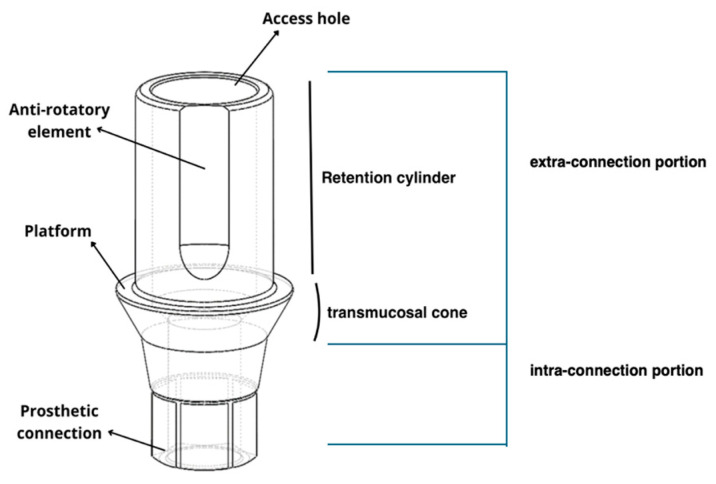
Graphic representation of a common Ti-base abutment design.

In order to avoid this type of complication, Ti bases were developed (Figure 1). These abutments are usually made of titanium grade 5 [4]. Their design consists of an intra-connection portion of the implant (thus mechanizing in this case two materials of similar elastic behavior), an extra connection with a conical transmucosal portion, which ends up on a seat platform on the crown retainer, and a coronal cylindrical portion, whose objective is the retention of the prosthesis via cementing. The result, therefore, is a complex abutment implant whose retention can be considered hybrid, i.e., cemented and screw-retained retention.

In terms of the portion of the abutment dedicated to retention, the height and angulation of the axial walls have been recognized in the prosthesis literature to play a role in its retention capacity through cementing (Sillingburg et al., 1997) [5]. The height of the prosthetic is one of the most important factors to guarantee proper cementation because if it increases, the contact surface will also increase and the cementation will therefore be stronger. Numerous studies show that the greater the height, the larger the retention [6,7,8]. In addition to the height, it has been demonstrated that when the convergence angle of the axial walls increases, the retention worsens [6]. At the same time, it is considered that a lower height and a greater convergence angle facilitate the insertion (with passive adjustment) of a structure that holds several implants and is connected to several abutments.

It is also considered that one of the functions of the abutment in general is that it helps to transmit the load more smoothly to the implant, the bone, and even the retention screws or the prosthetic structure [9,10,11]. In this regard, modifications of the design of the Ti base, which concern the width or angulation of its crown seat platform or the thickness of its walls, can affect the expected biomechanical behavior, which in turn can influence the occurrence of technical (decementation, fracture of the superstructures, chipping, etc.) or mechanical problems (loss of preload, fracture of the abutment, etc.). In general, it can be considered that there is little information in the scientific literature in this regard. A recent in vitro experimental study [12] considers that abutment with larger circumferential dimensions and robust structures could be associated with plastic deformation and bending of the implants. However, from the results of this study it is difficult to establish what is the width or thickness threshold that could cause these effects. It is also possible to find more information about the influence of the height of the transmucosal portion on peri-implant marginal bone loss, but this relationship seems to have more of a cause of biological width than purely mechanical [13].

Thus, this study was conducted with the main purpose of determining the influence of certain geometric features of the Ti base on the transfer and distribution of mechanical stress on the implant, the screw, and the Ti base itself and the resulting microdeformations in the supporting bone. In addition, the maximum load supported by the implant without exceeding the proportional elastic threshold of the implant was calculated for the different designs and these results were validated through an in vitro load test.

## 2. Materials and Methods

### 2.1. Finite Element Analysis

#### 2.1.1. Model Geometry

**Figure 2:**
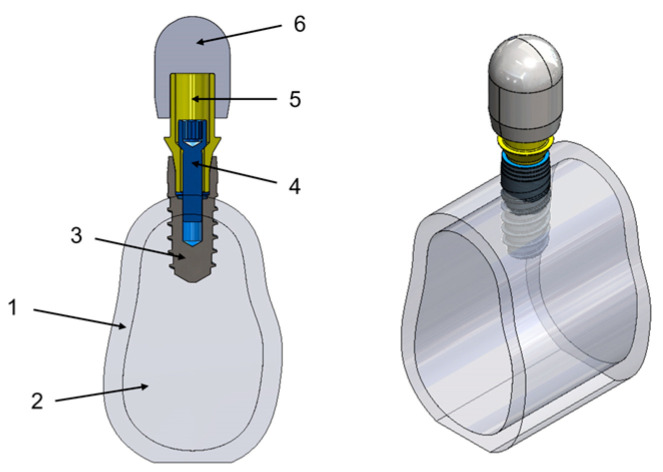
Three-dimensional design of the assembly of the dental implant system. Composed of cortical (1) and trabecular (2) bone, the dental implant (3), screw (4), Ti base (5), and hemispherical loading element (6). Own source.

A model formed using a segment of cortical and trabecular bone, together with the elements of the implant–prosthesis complex—implant, abutment (Ti base), screw, and hemispherical loading element (as shown in Figure 2)—was designed for use in finite element analysis.

For the modeling of the bone section, a design was produced following the basic structure of the adult mandibular bone. The model could be considered as type II according to the classification of Lekholm and Zarb (1985) [14]. For the measurements of the modeled bone segment, such as height (20 mm), dimensions found in the literature were used [15]. The width used in this model is based on the need for the implant to be surrounded in all directions by at least 1.5 mm of bone to reduce resorption [16]. The segment is 14 mm long, so it represents only a small fraction of the total mandible with a 1.5 mm thick cortical bone. The implant used for the investigation is the VEGA^®^ Implant (Klockner Implant System, Madrid, Spain), which is currently used in dental practice and has been studied in a similar way by several authors [17,18]. It is manufactured in grade 4 cold-worked (CW) titanium with dimensions of 10 mm in height and 4 mm in diameter. Although it is an internal connection, the bone-level implant was placed 3 mm above the bone level to simulate the worst-case condition. 

The screw connecting the implant to the Ti base was modeled with a head diameter of 2.2 mm and a total height of 8 mm.

In the case of the Ti base, the MEDPROTIBASE^®^ abutment (Klockner Implant System, Madrid, Spain), manufactured with grade 5 titanium (Ti6Al4V), was used as a base model and based on modifications to its design, the following eight specimens were established, which constitute the independent variables (Table 1):

- Width: 0.3 mm (narrow) and 0.5 mm (wide).
- Chamfer: 0° (NC) and 20° (C).
- Thickness: 0.3 mm (thin) and 0.4 mm (thick).

Finally, the prosthetic crown was modeled as a hemispherical element, as indicated in the ISO 14801 standard [19], corresponding to the Dynamic Loading Test for Endosseous Dental Implants. The main justification for this simplification was to be able to validate the FEA through an experimental physical analysis carried out under the precise conditions of the same standard. This element was produced from AISI 630 stainless steel, with a Young’s modulus of 197 Gpa, which is similar to that of zirconia (200 Gpa).

#### 2.1.2. Material Properties and Interface Conditions

The elastic properties, Young’s modulus, and Poisson’s ratio of the modeled materials were obtained from the literature and are listed in Table 2. In addition, the same table shows the ultimate tensile and yield strength values of grade 4 and 5 Ti, from which the implants and bases are commonly manufactured, respectively. All materials, including the supporting bone, were considered linearly elastic, homogeneous, and isotropic. The interface between the bone and the implant was assumed to be 100% ideal osseointegration. Likewise, a passive, effective fit without displacement between the implant and the modeled prosthetic portion (retention screws, Ti bases, and hemispheric element) was considered.

#### 2.1.3. Loads and Boundary Conditions

**Figure 3:**
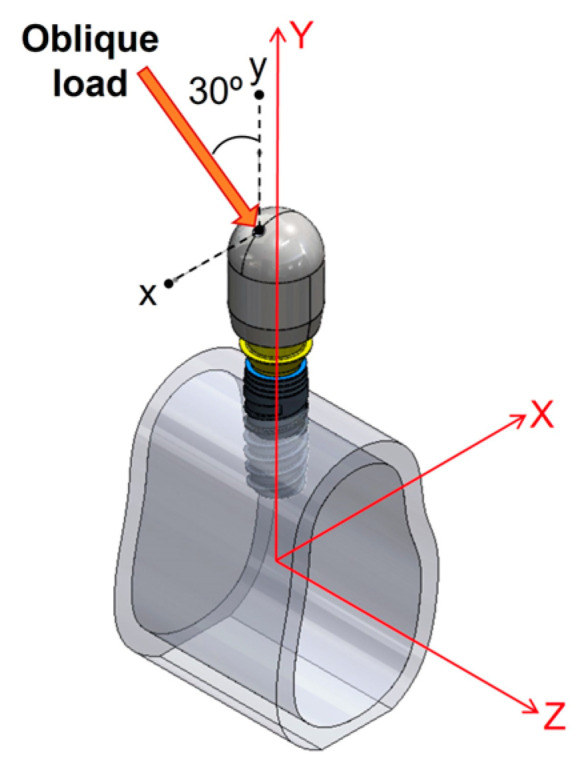
Scheme of the loading conditions applied.

The load simulated in the finite element analysis is schematized in Figure 3. A force of 360 N [20] was applied with an angulation of 30° with respect to the axial axis of the implant.

The sensitivity analysis showed that a mesh with 500,000 (or more) elements was sufficient to accurately represent the displacements and stresses. Therefore, it was decided that the maximum and minimum sizes of the elements of the meshes to be used would be 2 mm and 0.05 mm, respectively. The meshes obtained in the different models show variations associated with changes in geometry, which cause the number of elements to range from 653,698 to 490,748. Similarly, the number of nodes is also affected, with values varying between 68,858 and 49,081.

#### 2.1.4. Dependent Variables Analyzed

The results of the FEA are expressed with the maximum stress values (Mpa) according to the von Mises stress and its distribution for all modeled alloplastic components (Ti base, retention screw, and implant). However, for both the trabecular and cortical support bone, the resulting microdeformations (mm/mm) were measured, considering that these are involved in adaptive response processes. Finally, to determine which model would have the best performance in a simple and reliable way (considering only the information obtained in this study), a series of calculations was conducted to determine the maximum load that the assembly can withstand with each model before the resulting deformations on the implant were not proportional to the applied stress (proportional limit). In this case, it was considered that the critical component is the implant, so it was found that the maximum load that can be applied is the one that produces a maximum von Mises stress equal to the yield strength of grade 4 cold-worked (CW) titanium, with which the VEGA^®^ implants (Klockner Implant System, Madrid, Spain) used in the FEA and the experimental study are manufactured. The thermal treatment to which these implants are subjected generates improvements in the mechanical properties, with a yield strength of between 701 and 710 Mpa and an ultimate tensile strength of 832–851 Mpa when compared to those of conventional grade IV titanium, as displayed in Table 2. Once this information was obtained, the maximum load that was needed to be applied to reach these exact values for each model was calculated.

### 2.2. Experimental Physical Analysis

**Figure 4:**
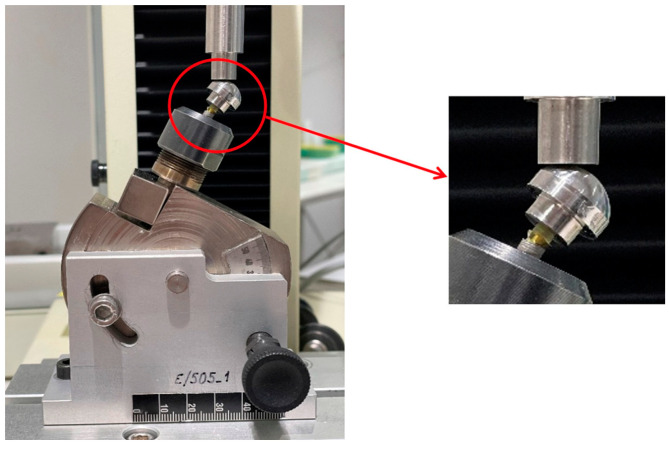
Setup for the bending test analysis. Own source.

To corroborate the results obtained with the finite element analysis regarding the maximum load produced by the plastic deformation of the implant, an experimental physical analysis of the W-T-NC model was performed. The specific choice of this model was based on the fact that it is an existing abutment on the market (MEDPROTIBASE^®^ abutment, Klockner Implant System, Madrid, Spain) and clinically used. The experiment was an adaptation of the ISO 14801 standard for static bending tests [19] (see Figure 4). The entire testing process was conducted at SOADCO S.L. facilities R&D&I laboratory (Escaldes Engordany, Andorra)

For this test, the Ti base (W-T-NC) was screwed into the implant with a torque of 30 N/cm according to the manufacturer’s recommendations. Both the VEGA implants and their corresponding Ti bases used in this experimental analysis are exact replicas, in all their characteristics, of those in clinical use and subjected to the same process of milling manufacturing, using computer numerical control, on EVO DECO turning machines (Tornos, Montier, Switzerland). The loading member, custom manufactured as a hemispherical element following the applied ISO standard, was then placed in the Ti base by friction. The entire set (implant –Ti base–hemispherical loading member) was placed on the load creep machine (Hounsfield H5KS, Tinius Olsen, Horsham, PA, USA), where it was held in place with a chuck/clamp with a torque of 5 N/m. The software was programmed with the following parameters: preload, 5 N; load, 1000 N; incremental speed, 1 mm/min; and drop limit, 10%. The test was carried out on a total of five specimens (N= 5). The Metrotest software v4.8 (Metrotec, Techlab Systems, Lezo, Spain) of the creep testing machine averaged the data and generated a single load–displacement graph. 

## 3. Results

### 3.1. Von Mises Stress on Implant, Ti Base, and Screw

It should be noted that the resulting maximum stress values transferred and their distribution, according to von Mises, are concentrated at the point opposite to the application of force on all the components of the system due to the simulated shift direction.

**Figure 5:**
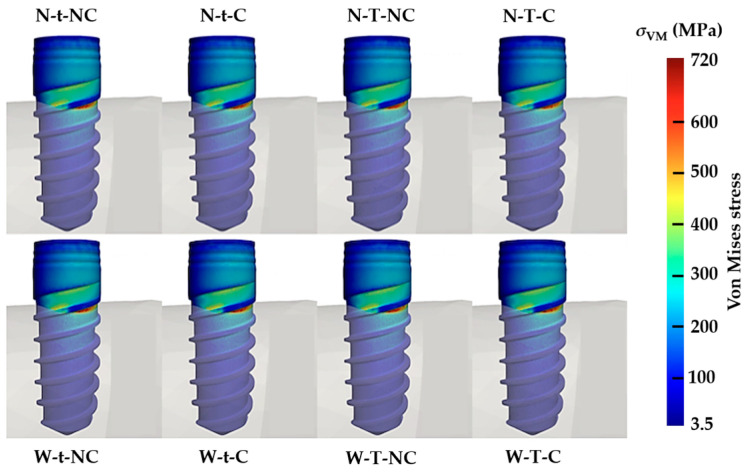
Numerical simulation representing the stresses according to the von Mises stress transferred to the implants, corresponding to the eight Ti-base designs analyzed.

On evaluation of the stress distribution colorimetry diagrams for the implants in Figure 5, it is observed that the areas of highest concentration occur at the first points of contact between the implant and the cortical bone. These results are consistent with those obtained in this type of analysis and are usually explained based on the differences between the elastic properties of the implant and the supporting bone. However, there are no variations in the distribution at the level of the implants in any of the eight Ti-base models studied.

**Figure 6:**
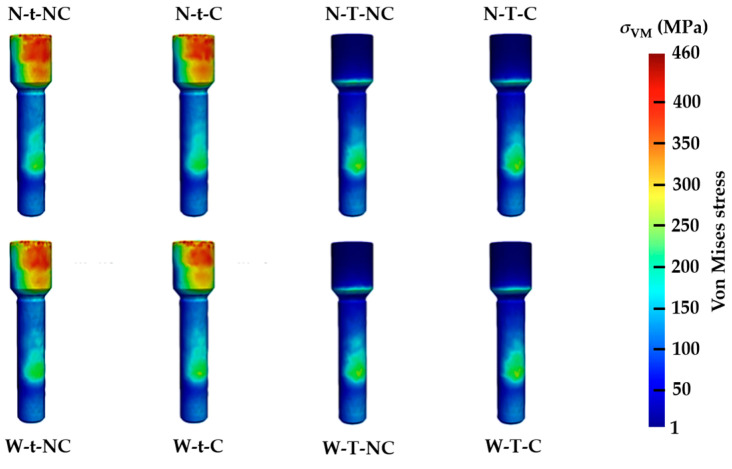
Numerical simulation representing the stresses according to von Mises transferred to the retaining screws, corresponding to the eight Ti-base designs analyzed.

**Figure 7:**
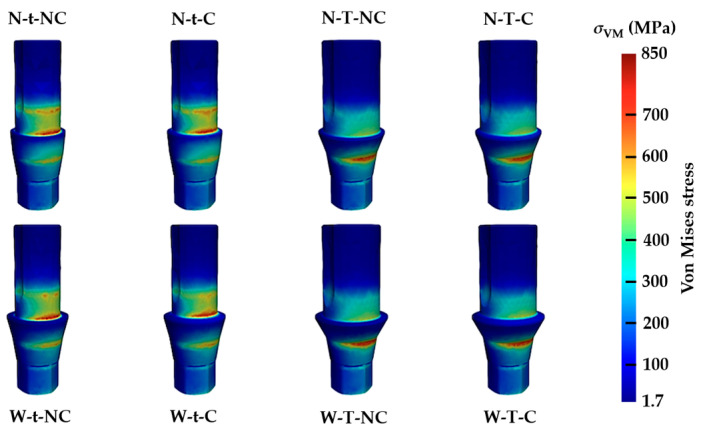
Numerical simulation representing the stresses according to von Mises transferred to the Ti bases, corresponding to the eight designs analyzed.

However, with regard to the retaining screws (Figure 6), it is evident that the most important stress distributions occur in relation to a single design variable of the Ti base: t = thin thickness. Likewise, this same variable (t = 0.3 mm and T = 0.4 mm) governed the differences in stress distribution in the Ti base (Figure 7). Indeed, the largest distribution of values for t occurs in the convergence between the axial wall and the column platform, and for T it occurs in the mechanization cone with the internal connection of the implant.

The maximum stress values of the eight different typologies according to von Mises at the level of the implant, retention screw, and Ti base are shown in Table 3. The first conclusion is that for all cases, the maximum values are always higher in the Ti base, then the implant, and finally the retention screw. The W-t-NC test model presents the highest tension at the level of the abutment and, at the same time, the lowest at the level of the implant. In fact, a relationship between the absence of chamfer (NC) and the appearance of higher levels of tension according to von Mises, at the level of the Ti base itself, can be described. However, it should be noted that the variable t (thickness) is clearly involved in the increase in the transfer of maximum stress, in this case at the level of the retention screw. These values are nearly double those of the four typologies of T thickness. This can have clinical repercussions if it is considered that the possibility of the fracture of a cylindrical element is intimately related to its radius.

### 3.2. Equivalent Strain on Cortical and Trabecular Bone

Taking into account that the adaptive responses of bones are influenced by the levels of deformation they endure when performing their function, the resulting microstrains (mm/mm) in the modeled cortical and trabecular bone were calculated.

**Figure 8:**
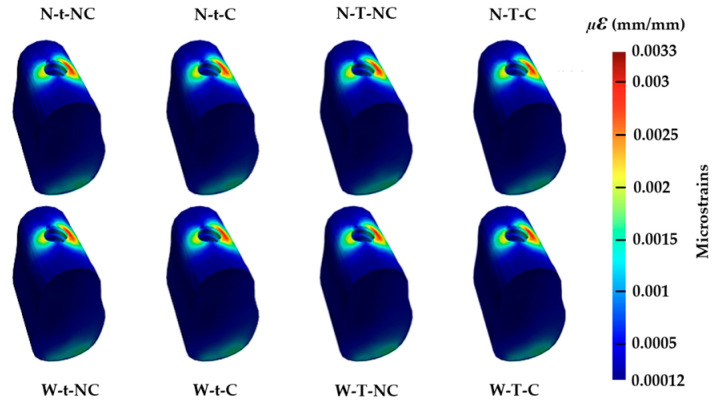

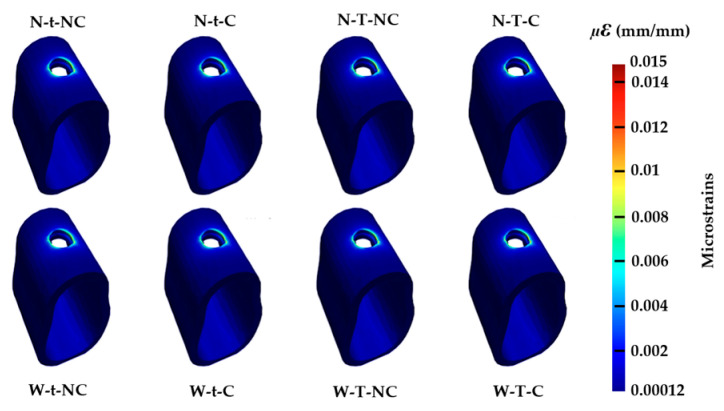
Microstrains on the cortical and trabecular bone for the different models. Own source.

Again, the greatest strain received by the cortical bone was detected in the crestal area, i.e., in the first contact points between the bone and the implant (Figure 8). In the case of the trabecular bone, the largest strains were encountered at its margin, around the intersection with the implant (Figure 8). It should be noted that, as in the case of implants, it is not possible to describe the different types of resulting microdeformations for the eight types of Ti bases in this study.

When analyzing the results of the maximum values of microdeformation (Table 4) at the level of the bone (cortical and trabecular), it is not possible to observe large differences across Ti base types or individual design factors. However, for all cases, the values of microstrains are higher in the cortical bone than in the trabecular bone.

### 3.3. Maximum Load Comparison

Table 5 shows the results obtained through the finite element analysis, which express the maximum load values on each of the Ti-base designs under study. These values are related to the stresses that exceed the proportional limit between the applied load and the resulting deformations of the implant VEGA.

The results show there are not significant differences in the maximum load that the models can withstand. Nevertheless, the best performance is exhibited by W-t-NC, with a maximum of 370 *N*. The model related to the lowest maximum stress value was W-T-C, again with thickness influencing this variable.

### 3.4. Comparison with Experimental Results

To validate the results for the maximum load of the FEA, an experimental in vitro test was carried out in accordance with the ISO 14801 standard. It was applied to the W-T-NC model, which corresponds to a control, since it is a typology marketed and used in clinics and also presented lower maximum load values (lower second) in the finite element test compared to the rest of the typologies.

**Figure 9:**
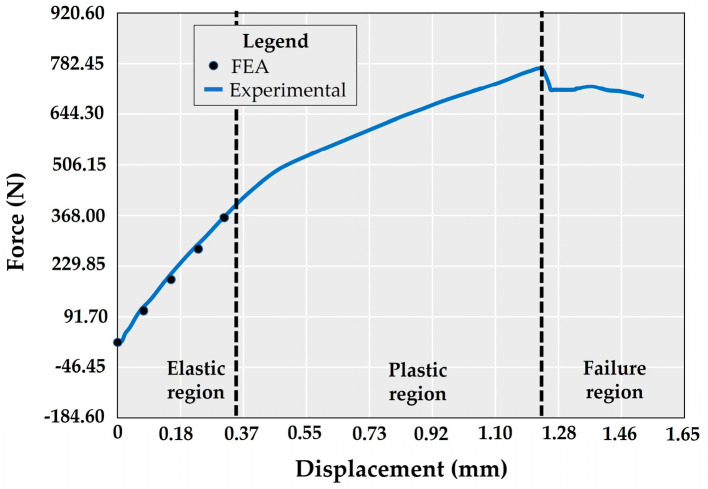
Comparison of the force–displacement curve behavior of the W-T-NC model. Blue line: experimental bending test. Black dots: present finite element.

The load–displacement graph shows the average strain curve of the five specimens tested in vitro (Figure 9). From this, we can find the load and deformation at which the yield strength and ultimate tensile strength are reached and, eventually, the implant will fail. On the other hand, in the different specimens evaluated, it was found that the proportional limit is reached at a load level of 383.216 N on average (sd 10.33 N).

In addition, the finite element results were plotted on the same graph. For this, the value at which the proportional limit was exceeded in the real test was used, so the indicated model (W-T-NC) was simulated under loads of 25%, 50%, 75%, and 100% of 380 N. In this way, it was possible to produce a load–displacement graph that could be compared with the experimental one. It can be observed that both results are very similar in the elastic region and before entering the plastic deformation state. It is important to note that, although the test was conducted with a maximum load of 380 N, the finite element model failed under loads above 362 N (Table 5). A Root-Mean-Square Error (RMSE) analysis was performed to determine the statistical difference between the experimental test and the finite element simulation. The results of this RMSE show a difference of 0.45% between the two tests.

## 4. Discussion

The present study used FEA and aimed to determine the influence of different aspects of the Ti-base design on its biomechanical behavior in relation to variables such as tension and its distribution in the abutments, retention screws, and implants and the resulting microformations in the bone.

Considering the results around the implant, the first appreciable feature is that for all models, the stress distributions are practically identical, regardless of the Ti-base model analyzed. The stresses are concentrated in the area closest to the bone, also called the implant–bone intersection. This is a fact supported by the literature [6,21,22] and is already assumed to be normal behavior for the implant under non-axial loads. This behavior is due to what is known as the “Law of the Lever”, where a concentration of stress is produced at the first point of contact between the lever (in this case the implant) and the supporting point (in this case the cortical bone). In general, it is considered that this behavior is highly influenced by the different elastic properties of the materials in contact (titanium and bone). The same result can be observed in studies of the same methodology and similar design but in which the implant is placed at bone level [18]. In addition, a finite element analysis was carried out to determine the maximum load values on each model that could cause the implant to exceed its proportional limits in the resulting deformations. Although differences have been found between typologies, the average value is 364.5 N and the difference between the designs with the highest (W-t-NC) and lowest (W-T-C) values is only 16 N. This difference value constitutes only 4.38% of the mean value found, and although it is a low percentage value, it could have an influence on possible mechanical failures, especially when an unfavorable direction of force application is added to this magnitude.

The comparison with the results of the in vitro study of static load creep shows that these values are expected for the limit of the elastic behavior of the implant and, ultimately, that the finite element model applied has had an effective design in terms of obtaining data of biomechanical interest. Although the initial choice of the load magnitude of the tests (360 N) was based on data from the literature [20], it cannot be assumed that all the force will be concentrated in a single crown and a single implant. In this regard, Watanabe and collaborators [23] (2008), in their observational study, described a chewing force of greater magnitude ($\bar{x}$1024 N, sd 410 N) in patients with natural teeth but with a certain distribution for each of the teeth in terms of magnitude and direction. In that sense, the tooth with the greatest force applied is the second molar, with up to about 250 N on average, but with a vertical direction of load and therefore a more favorable moment than that applied in our trial. In short, in view of the results, it could be said that any of the eight Ti-base models analyzed can be sufficiently protective against mechanical implant failure.

As expected, the area of cortical bone that undergoes the greatest strain is the crestal area that is in direct contact with the implant. A similar result has been found in studies carried out by other research groups [17,18]. In the trabecular bone, on the other hand, the strain is localized around the implant but not so concentrated at the intersection. The results found in the cortical bone stand out positively, since they show a maximum deformation of around 0.014 (mm/mm). These values are in good agreement with the results reported in the study by Oliveira and colleagues (2020) [17], where three different cortical bone thicknesses (0.5, 1, and 2 mm) were analyzed. In a model composed of the VEGA^®^ implant (Klockner Implant System, Madrid, Spain) and bone of 850 HU (Houndsfield Units), the results obtained with different thicknesses vary between 0.0111 and 0.0117 (mm/mm), so they can be considered comparable to those obtained in our study. Something similar occurs when the present results are compared with those reported in another study, with the difference that in this case both the implant model and the thickness of the cortical bone are the same as those in the present work [18]. On the other hand, the results obtained for the trabecular bone do not seem to coincide with those of the studies reviewed. In this study, maximum strain values of around 0.0035 (mm/mm) were obtained, but they clearly differ from the 0.025 (mm/mm) found in [18]. However, as a summary of the objectives of the study, no relationship could be established between the Ti-base design factors and different microdeformations of the supporting bone.

Focusing on the aspects of the modifications of the design of the Ti base, it can also be observed how, in all the models, the presence of the chamfer causes a large variation in the stresses supported, especially at the level of the abutment itself. These results are in good agreement with other reports that showed that using a chamfer in the platform will reduce the stresses [24]. In the case of platform width, the results show how, in three of the four cases, the stresses are higher in the models with the wide platform. Despite this, the greatest biomechanical influences seem to be concentrated on a single aspect of the design: the thickness of the Ti base. The results show that a smaller thickness (0.3 mm) is associated with a less favorable stress distribution and a transfer of maximum stress values according to greater von Mises to the retention screws. This circumstance may have the greatest clinical implications, since the technical complications of the retaining screws (loss of preload and fracture) are more frequent than those of the abutments or implants, as shown by various systematic reviews in the literature [25,26]. Considering our results, a Ti base with a greater wall thickness could limit the stresses on the retention screws before the application of load and therefore prevent complications. However, it must be considered that in our study, in order to adapt to the standard for a static bending test, we used a steel hemispherical element instead of a zirconia crown, which would be logical for this type of abutment. In this regard, the width and angulation of the platform can greatly influence the behavior of the zirconia crown that it retains if it is intended to work with resulting compression and sufficient surfaces of tension dissipation. This aspect may be of interest if it is considered that the most frequent technical complication in zirconia prostheses is the fracture or detachment of esthetic ceramic coatings [3].

A recent in vitro experimental study [2] compared the mechanical behavior of six different types of Ti-bases under fatigue and thermocycling tests. In general, the authors did not find differences between the six typologies, but they did in a later fracture load test. This study, however, describes the differences in the Ti-base models used based on the transmucosal portion (gingival height) and the retention cylinder portion (prosthetic height), two factors that we have not analyzed, which therefore makes it difficult to discuss in relation to our results. Although in the future the possible influence of the prosthetic height on biomechanical behavior should continue to be studied, it is necessary to consider that this height has a great influence on the retention by cementation of the crown and that its design must at all times take this aspect into consideration. 

It is essential to recognize that this study is not without limitations, since it was necessary to assume a series of simplifications to carry out the analysis, such as the consideration of the isotropy and the linear elasticity of the modeled elements (especially the bone), the assumption of 100% bone integration, or the absence of displacement before the application of load to the mechanized elements. However, it is also true that these types of simplification are present in most of the FEA studies present in the scientific literature [27,28]. Additionally, as we have mentioned above, the prosthetic crown used in the test was modeled as a hemispherical element, following the ISO 14801 standard, in order to correspond to the Dynamic Loading Test for Endosseous Dental Implants and to be able to more effectively compare the results of the FEA with those of the in vitro load test. However, taking into consideration the principle of Saint-Venant, the simplified shape of the crown should not affect the load resulting from tension.

## 5. Conclusions

The results of the FEA study show that the design modifications of the Ti base influence the biomechanical behavior and, ultimately, the way in which the stress is transferred to the entire prosthesis–implant–bone system.

If the results of the in vitro validation are considered, then all the models analyzed would be able to prevent the mechanical failure of the implant in the face of normal masticatory load conditions.

However, it can be concluded that the features that give the Ti base a better capability to distribute stresses are the chamfer (20°), a thick wall (0.4 mm), and a narrow platform (0.3 mm). In addition, a greater thickness should be used for the mechanical protection of one of the possible weak points of the system, such as the retention screw. These specifications, although they require clinical validation, should be taken into consideration in future designs of this type of abutment.

This study constitutes the beginning of a project for our research group, to which future projects will be added, that will try to determine the most effective method of cementing bonding zirconia structures on the Ti base, the influence of alumina blasting on its surface in relationship with the residual surface stress and corrosion tendency, and the adjustments of the inclination of its axial walls to favor passive fit.

In short, the design of the Ti base is a key factor in prosthetic retention, passive fit, and mechanical behavior, so a greater number of both experimental and in vitro studies that explore the most efficient possibilities is desirable. 

## Figures and Tables

**Table 1:** Specific variables of the Ti base’s geometry and model names.

Width (mm)	Thickness (mm)	Chamfer	Model
0.3	0.3	no	N-t-NC
0.3	0.3	yes	N-t-C
0.3	0.4	no	N-T-NC
0.3	0.4	yes	N-T-C
0.5	0.3	no	W-t-NC
0.5	0.3	yes	W-t-C
0.5	0.4	no	W-T-NC
0.5	0.4	yes	W-T-C

**Table 2:** Elastic properties of the models’ materials.

Element	Material	Young’s Modulus (GPa)	Poisson Ratio	Ultimate Tensile Strength (MPa)	Yield Strength (MPa)
Ti Base	Ti grade 5	114	0.33	825–860	760–795
Implant	Ti grade 4	107	0.33	240–550	170–483
Retention Screw	Ti grade 5	114	0.33	825–860	760–795
Hemispheric Element	Stainless steel AISI 360	197	0.27		
Cortical Bone		11	0.3		
Spongy Bone		1	0.3		

**Table 3:** Von Mises stress values for the implant, Ti base, and screw in the different models.

Model	σ_VM_ Implant [MPa]	σ_VM_ Ti Base [MPa]	σ_VM_ Screw [MPa]
N-t-NC	693	825	459
N-t-C	698	785	464
N-T-NC	697	774	255
N-T-C	701	746	255
W-t-NC	690	846	425
W-t-C	705	743	484
W-T-NC	707	822	250
W-T-C	723	791	257

**Table 4:** Equivalent strain values for cortical and trabecular bone on the different models.

Model	µԐ_EQ_ Cortical (mm/mm)	µԐ_EQ_ Trabecular (mm/mm)
N-t-NC	0.015	0.0032
N-t-C	0.015	0.0031
N-T-NC	0.014	0.0032
N-T-C	0.014	0.0033
W-t-NC	0.014	0.0031
W-t-C	0.015	0.0032
W-T-NC	0.014	0.0032
W-T-C	0.014	0.0031

**Table 5:** Maximum load supported by the implant without exceeding the proportional limit of the titanium grade IV CW of the Klockner Vega implants. Stresses in the screw and Ti base and equivalent strains at the bone are also shown.

Model	Yield Strength σ_VM_ Implant (MPa)	σ_VM_ Screw (MPa)	σ_VM_ Ti Base (MPa)	Max. Load (N)
N-t-NC	710	470	846	369
N-t-C	710	472	798	366
N-T-NC	710	260	789	367
N-T-C	710	259	756	365
W-t-NC	710	437	870	370
W-t-C	710	488	749	363
W-T-NC	710	251	827	362
W-T-C	710	253	778	354

## Data Availability

The original contributions presented in the study are included in the article, further inquiries can be directed to the corresponding author.

## References

[1] Ramalho I., Witek L., Coelho P.G., Bergamo E., Pegoraro L.F., Bonfante E.A. (2020). Influence of abutment fabrication on 3D fit at the implant-abutment connection. Int. J. Prosthodont..

[2] Mühlemann S., Hjerppe J., Hämmerle C.H.F., Thoma D.S. (2021). Production time, effectiveness and costs of additive and subtractive computer-aided manufacturing (CAM) of implant prostheses: A systematic review. Clin. Oral Implants Res..

[3] Sailer I., Strasding M., Valente N.A., Zwahlen M., Liu S., Pjetursson B.E. (2018). A systematic review of the survival and complication rates of zirconia-ceramic and metal-ceramic multiple-unit fixed dental prostheses. Clin. Oral Implants Res..

[4] Edelhoff D., Beuer F., Weber V., Johnen C. (2008). HIP zirconia fixed partial dentures—Clinical results after 3 years of clinical service. Quintessence Int..

[5] Shillingburg H.T., Hobo S., Whitsett L.D., Jacobi R., Brackett S.E. (1997). Fundamentals of Fixed Prosthodontics.

[6] Choi H.W., Park Y.S., Chung S.H., Jung M.H., Moon W., Rhee S.H. (2017). Comparison of mechanical and biological properties of zirconia and titanium alloy orthodontic microimplants. Korean J. Orthod..

[7] Saber F.S., Abolfazli N., Nuroloyuni S., Khodabakhsh S., Mehran B., Nahidi R. (2012). Effect of Abutment Height on Retention of Single Cement-retained, Wide-and Narrow-platform Implant-supported Restorations. J. Dent. Res. Clin. Prospects..

[8] Emms M., Tredwin C.J., Setchell D.J., Moles D.R. (2007). The effects of abutment wall height, platform size, and screw access channel filling method on resistance to dislodgement of cement-retained, implant-supported restorations. J. Prosthodont..

[9] Turkoglu P., Kose A., Sen D. (2019). Abutment Selection for Anterior Implant-Supported Restorations. An Update of Dental Implantology and Biomaterials.

[10] Taheri M., Akbari S., Shamshiri A.R., Shayesteh Y.S. (2020). Marginal bone loss around bone-level and tissue-level implants: A systematic review and meta-analysis. Ann. Anat..

[11] Kumar V.V., Sagheb K., Kämmerer P.W., Al-Nawas B., Wagner W. (2014). Retrospective Clinical Study of Marginal Bone Level Changes with Two Different Screw-Implant Types: Comparison Between Tissue Level (TE) and Bone Level (BL) Implant. J. Maxillofac. Oral Surg..

[12] Karasan D., Pitta J., Zarauz C., Strasding M., Liu X., Fehmer V., Sailer I. (2023). The influence of titanium-base abutment geometry and height on mechanical stability of implant-supported single crowns. Clin. Oral Implants Res..

[13] Galindo-Moreno P., León-Cano A., Ortega-Oller I., Monje A., Suárez F., ÓValle F., Spinato S., Catena A. (2014). Prosthetic Abutment Height is a Key Factor in Peri-implant Marginal Bone Loss. J. Dent. Res..

[14] Brånemark P.I., Zarb G.A., Albrektsson T. (1985). Tissue integrated Prostheses: Osseointegration in Clinical Dentistry.

[15] Ural Ç., Bereket C., Şener I., Aktan A.M., Akpinar Y.Z. (2011). Bone height measurement of maxillary and mandibular bones in panoramic radiographs of edentulous patients. J. Clin. Exp. Dent..

[16] Monje A., Chappuis V., Monje F., Muñoz F., Wang H.L., Urban I. (2019). The Critical Peri-implant Buccal Bone Wall Thickness Revisited: An Experimental Study in the Beagle Dog. Int. J. Oral Maxillofac. Implants..

[17] Oliveira H., Velasco A.B., Ríos-Santos J.V., Lasheras F.S., Lemos B.F., Gil F.J. (2020). Effect of different implant designs on strain and stress distribution under non-axial loading: A three-dimensional finite element analysis. Int. J. Environ. Res. Public Health.

[18] Gil J., Sandino C., Cerrolaza M., Pérez R., Herrero-Climent M., Rios-Carrasco B., Rios-Santos J.V., Brizuela A. (2022). Influence of Bone- Level Dental Implants Placement and of Cortical Thickness on Osseointegration: In Silico and In Vivo Analyses. J. Clin. Med..

[19] (2016). Dentistry-Implants-Dynamic Loading Test for Endosseous Dental Implants.

[20] Shimada A., Yamabe Y., Torisu T., Baad-Hansen L., Murata H., Svensson P. (2012). Measurement of dynamic bite force during mastication. J. Oral Rehabil..

[21] Wang T., Wang L., Lu Q., Fan Z. (2021). Influence of anodized titanium abutments on the esthetics of the peri-implant soft tissue: A clinical study. J. Prosthet. Dent..

[22] Choi K.H., Son K.B., Lee D.H., Lee K.B. (2018). Influence of abutment height and convergence angle on the retrievability of cement- retained implant prostheses with a lingual slot. J. Adv. Prosthodont..

[23] Watanabe M., Hattori Y., Satoh C. (2005). Biological and biomechanical perspectives of normal dental occlusion. International Congress Series.

[24] Mieda M., Atsuta I., Matsushita Y., Morita T., Ayukawa Y., Tsukiyama Y. (2018). The effective design of zirconia coping on titanium base in dental implant superstructure. Dent. Mater..

[25] Omori Y., Lang N.P., Botticelli D., Papageorgiou S.N., Baba S. (2020). Biological and mechanical complications of angulated abutments connected to fixed dental prostheses: A systematic review with meta-analysis. J. Oral Rehabil..

[26] Bidra A.S., Rungruanganunt P. (2013). Clinical outcomes of implant abutments in the anterior region: A systematic review. J. Esthet. Restor. Dent..

[27] Brizuela-Velasco A., Pérez-Pevida E., Jiménez-Garrudo A., Gil-Mur F.J., Manero J.M., Punset-Fuste M., Chávarri-Prado D., Diéguez-Pereira M., Monticelli F. (2017). Mechanical Characterisation and Biomechanical and Biological Behaviours of Ti-Zr Binary-Alloy Dental Implants. Biomed. Res. Int..

[28] Alvarez-Arenal A., Gonzalez-Gonzalez I., deLlanos-Lanchares H., Brizuela-Velasco A., Martin-Fernandez E., Ellacuria-Echebarria J. (2017). Influence of Implant Positions and Occlusal Forces on Peri-Implant Bone Stress in Mandibular Two-Implant Overdentures: A 3-Dimensional Finite Element Analysis. J. Oral Implantol..

